# Can Serum Iron Concentrations in Early Healthy Pregnancy Be Risk Marker of Pregnancy-Induced Hypertension?

**DOI:** 10.3390/nu11051086

**Published:** 2019-05-16

**Authors:** Małgorzata Lewandowska, Stefan Sajdak, Jan Lubiński

**Affiliations:** 1 Division of Gynecological Surgery, Poznań University of Medical Sciences, 60-535 Poznań, Poland; ssajdak@ump.edu.pl; 2 Department of Genetics and Pathology, International Hereditary Cancer Center, Pomeranian Medical University, 71-252 Szczecin, Poland; lubinski@pum.edu.pl

**Keywords:** pregnancy, hypertension, iron, risk, preeclampsia, micronutrient, prospective study

## Abstract

The aim of this study was to assess the relationship between serum iron concentrations in early healthy pregnancy and the risk of pregnancy-induced hypertension. The data comes from our prospective cohort study in which we recruited healthy women in week 10–14 of single pregnancy. We examined a study group (*n* = 121) consisting of women subsequently developing pregnancy-induced hypertension and a control group (*n* = 363) of matched women remaining normotensive. We measured iron concentrations in the serum collected in 10–14 gestational week, using the ICP-MS technique (mass spectrometry with inductively coupled plasma). The odds ratios of the disease (95% confidence intervals) for iron concentrations were assessed in multivariate logistic regression. We found that the mean microelement concentration was lower in the case group compared to normotensive controls (*p* = 0.011). Women in the lowest quartile of iron (≤801.20 µg/L) had a 2.19-fold increase in pregnancy-induced hypertension risk compared with women in the highest quartile (>1211.75 µg/L) (odds ratio (OR) = 2.19; 95% CI: 1.24–3.88; *p* = 0.007). This result was sustained after adjusted for all the accepted confounders. Women in the higher Q_2_ quartile (801.20–982.33 µg/L) had a 17% lower risk, compared with those in the highest quartile (OR = 0.83; 95% CI: 0.65–2.32; *p* = 0.519).

## 1. Introduction

An adequate micronutrient status is required to ensure optimal pregnancy development [1,2,3]. Iron status in pregnant women may arouse interest, due to its involvement in many biochemical processes, including oxidative balance. However, it has not yet been established whether iron concentrations can be a marker of the risk of pregnancy-induced hypertension in which oxidative stress plays a key role. Iron deficiency is common in pregnancy. Iron deficiency can lead, among others, to decreased hemoglobin levels and reduced immunity [4,5,6,7,8]. In pregnancy, iron deficiency correlates with preterm delivery and low birthweight [4,5,6,7,8]. In the other hand, high level of hemoglobin in women supplemented with iron was associated with an increase in the frequency of adverse effects, including hypertension disorders in pregnancy [4,5,6,7,8]. Pregnancy induced hypertension (PIH) affects an average of 5-10% of pregnant women, includes isolated gestational hypertension (GH) and preeclampsia (PE), and increases morbidity and mortality of mothers and fetuses [9,10]. Many clinical risk factors have been identified include, among others: Chronic hypertension, pre-pregnancy diabetes, maternal age, parity, methods of assisting reproduction, and obesity [9,11]. However, until now, the markers for predicting this disease have not been clearly established.

Iron has important metabolic functions in cells, but it can be biochemically dangerous [4,5,6,7,8]. Iron is involved, inter alia, in oxygen transport, in the production of ATP, in the synthesis of nucleic acids, in the maintenance of mitochondrial fusion and protection of cellular structures against oxidative damage, in the transcriptional activation pathway of hypoxia-inducible factor (HIF) playing a significant role in the development of the placenta, in the growth and proliferation of cells, as well as in the activity of numerous enzymes [12]. At the same time, iron is a transition element; it is a catalyst for the Fenton and Haber-Weiss reactions, and when in excess, it can promote oxidative stress and damage to endothelial cells [13].

Oxidative stress is a recognized element of the pathogenesis of pregnancy-induced hypertension (PIH) [11]. The etiology of this disease is not fully understood, but it has been found that abnormal invasion of trophoblasts in the walls of the spiral arteries (between 6-18 week of pregnancy) results in their insufficient re-modelling, which leads to utero-placental high-resistance circulation. This results in hypoxia and intensification of the production of reactive oxygen species (ROS), affecting apoptosis and the immune system, and intensification of the inflammatory response. The cascade of processes in the maternal circulation system is activated and leads to damage of the vascular endothelium. The disturbance in endothelial homeostasis results, among others, in pro-inflammatory and pro-thrombotic tendency, in a vasoconstrictive and in an increase in blood pressure [9,10,11,14,15]. During normal pregnancy, oxidative stress in the placenta is present (it is necessary to obtain normal cell function). Oxygen free radical levels are increased under conditions of hypoxia. Iron plays a catalyzing role in the production of reactive oxygen species in the Fenton and Haber-Weiss reactions [13,15].

In the world literature, studies which assess the association of iron concentrations with pregnancy-induced hypertension are available, but these are mainly retrospective studies regarding women with already developed disease. As shown by meta-analyses, in most retrospective studies, statistically significantly higher serum iron concentrations were found in women with preeclampsia compared to normotensive women [16], but there are also contrary results [17]. However, iron concentrations, in women who have already developed the disease, may be a result of existing disorders.

Only prospective studies, evaluating iron status in early pregnancy, can explain whether iron concentrations may be a risk marker for pregnancy-induced hypertension. In the world’s literature, we have found few prospective studies, and among those, the results are divergent and the research methodologies differ [18,19].

The aim of our study was to assess the relationship between the maternal serum iron concentration in early healthy pregnancy and the risk of pregnancy-induced hypertension. To our knowledge, this is the first single-center prospective cohort study for iron concentrations conducted in so many cases of this disease.

## 2. Materials and Methods

The report on this study was carried out in accordance with the guidelines for study designs (from Equator network).

### 2.1. Ethics Statement

The study was conducted in accordance with the Helsinki Declaration and was approved by the Bioethics Committee of the Medical University of Poznan, Poland, under number 769/15. All the pregnant volunteers signed the Test Information Form and the Informed Consent Form before submitting a blood sample.

### 2.2. Participants

The data comes from our prospective cohort study in which we recruited 1300 women. We conducted this study at the University Hospital in Poznan, Poland (it is third-degree reference center, with 6–8 thousand births a year). The recruitment was covered pregnant women taking typical laboratory tests. The recruitment, observation, and analysis of results were conducted in 2015–2016, 2016–2017, and 2017–2019, respectively.

Inclusion criteria covered: Women of white Polish (European) descent from the Wielkopolska region, aged 18–45 years at conception, healthy, in 10 (+0)–14 (+6) week of healthy single pregnancy (with subsequent delivery of a phenotypically normal child ≥25 gestational week), no chronic diseases beyond being overweight or obese (no chronic hypertension, kidney, and liver diseases, and pre-pregnancy diabetes mellitus, no immunological and inflammatory diseases or thromboembolism), no chronic or active infection, using normal diet. The use of multivitamin preparations was not a condition of inclusion in the study.

In the present study, we examined cohort of 484 women: The study group (*n* = 121) consisting of women subsequently developing pregnancy-induced hypertension (106 cases of gestational hypertension and 15 cases of preeclampsia); the control group (*n* = 363) consisting of women who remained normotensive.

The minimum sample size was calculated using the formula for a single proportion. For the proportion of pregnancy-induced hypertension *p* = 10% (based on the literature) and the margin error d = 0.02, the estimated sample size was 864 (confidence intervals 95%, α = 5%). For the proportion *p* = 14% (based on our population) and the margin error d = 0.03, the estimated sample size was 514.

**Figure 1:**
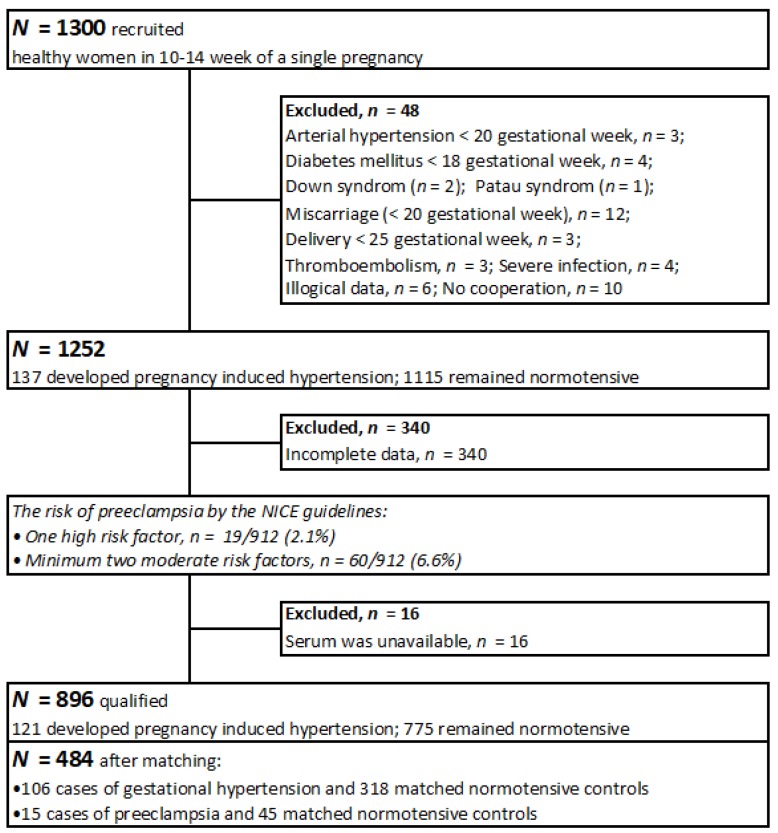
The recruitment scheme.

The recruitment scheme is presented in Figure 1.

### 2.3. Method and Data Collection

Data (obstetrical and gynecological histories, concurrent diseases, socio-demographic characteristics, medications and vitamins, smoking, alcohol consumption, family medical histories) were collected using a personal questionnaire during the recruitment. The women filled out the questionnaires on their own to avoid any suggestions being made on the part of the interviewers.

The women were observed up to 12 weeks after parturition. We contacted the participants by telephone or e-mail. Pregnancy outcomes were taken from the Medical Records; some information was also passed on by the participants themselves. The information was verified during the observation. After the observation, we excluded the women who did not meet all inclusion criteria (*n* = 48), and women whose responses in the questionnaires were incomplete and during the observation were unable to supplement this data (*n* = 340).

The use of folic acid in the first trimester and multivitamins in II- III trimester and the weight before pregnancy were declared by the participants themselves. The normal pre-pregnancy body mass index (BMI) was defined as 18.5–24.99 kg/m^2^. Gestational weight gain was defined as the difference between the weight measured before delivery (from medical records) and the pre-pregnancy weight. All women declared no alcohol in pregnancy.

The data included the following maternal education level categories: Elementary, vocational, secondary, or higher. The financial status of the study participants was assessed according to a 5 Lickert’s scale, on the basis of an answer to the question: “Is your financial situation (in your household) good enough to meet your needs?” The responses were classified in the following way: (1) definitely NO; (2) rather NO; (3) hard to say; (4) rather YES; (5) definitely YES. In this survey we distinguished: lower financial status (1 and 2), medium (3), and higher (4 and 5). Data concerning the place of residence, included the following categories: Countryside; small town (<50 thousand inhabitants); big city.

Pregnancy-induced hypertension was defined in accordance with the national guidelines (2015), convergent with a new definition of preeclampsia [9]. Gestational hypertension was defined as “arterial pressure equal to, and higher, than 140/90 mmHg (on two occasions, at least 4 h apart, with an oscillometric device) developed de novo after the 20th week of pregnancy, receding up to 12 weeks after delivery; and was diagnosed if no other disturbance was found”. Preeclampsia was defined as “arterial pressure equal to and higher than 140/90 mmHg developed de novo after the 20th week of pregnancy, receding up to 12 weeks after delivery; and was diagnosed when any of the following appeared de novo: Proteinuria (≥300 mg/day or ≥0.3 g/L, protein/creatinine ratio ≥0.3; 1+ in the strip test), thrombocytopenia < 100 G/L, worsening of renal function (creatinine >1.1 mg/dL or doubling of creatinine in chronic kidney disease); damage to the liver function (increase in ALAT and ASPAT twice the norm); pulmonary edema; symptoms from the central nervous system; blurred vision”. In our population, only proteinuria (≥300 mg/L) occurred in the cases of preeclampsia. IUGR (Intrauterine Growth Restriction) was not a criteria of diagnosis.

Clinical risk factors of pregnancy-induced hypertension [9,11] and influencing factors related to the concentration of iron [20,21] have been identified based on literature data.

The risk of preeclampsia in the population was assessed according to the NICE (National Institute for Health and Clinical Excellence) guidelines (Figure 1) [22].

### 2.4. Serum Iron Determination

We measured iron concentration in maternal serum. The serum came from samples taken during recruitment in 10–14 (+6) week of pregnancy (from women declaring themselves to be fasting). Blood from cases and controls was collected with Sarstedt Monovette system (Sarstedt, Nümbrecht, Germany) using Serum Z/7.5 mL tubes. Collected blood was incubated at room temperature for at least 30 min., but no longer than 2 h to clot and then was centrifuged at 1300× *g* for 12 min. After that, serum was transferred into cryo vials and placed into freezer at −80 °C. Patient sera were stored at −80 °C until analysis. At the day of analysis sera were thawed, vortexed and centrifuged at 5000× *g* for 5 min before iron determination.

To measure concentrations of iron, we used the method of mass spectrometry with inductively coupled plasma (ICP-MS). The samples of total iron determination was performed using ICP mass spectrometer NexION 350D (PerkinElmer, Shelton, USA). Before each analytical run, the instrument was tuned to achieve manufacturers’ criteria. Methane was used as a reaction gas. Technical details are available on request.

The spectrometer was calibrated using an external calibration technique. Calibration standards were prepared from 10 µg/mL Multi-Element Calibration Standard 3 (PerkinElmer, Shelton, USA) by diluting with blank reagent to the final concentration of 1, 5, 10 and 50 µg/L. Correlation coefficients for calibration curves were always greater than 0.999. Analysis protocol assumed 100 fold dilution of serum in blank reagent. Blank reagent consists of 10 mL of 65% Suprapur Grade nitric acid (Merck, Darmstadt, Germany), 0.20 mL of Triton X-100 (PerkinElmer, Shelton, USA) filled to the mark of 1 L flask with class I deionized water (Merck Millipore, Darmstadt, Germany). Germanium isotope (Ge74) was set as internal standard.

Accuracy and precision of measurements were tested, using a certified reference material (CRM), Clincheck Plasmonorm Serum Trace Elements Level 1 (Recipe, Munich, Germany). Additionally, internal quality control samples were measured during analysis. General precision was lower than 5% RSD. The final concentration included a dilution factor and coefficient which was the mean value of two flanking certified reference materials concentrations divided by mean concentration determined by the manufacturer of CRM.

Two measurements of iron were not obtained.

Other microelement (Zn, Cu, Se) determination details are available on request.

### 2.5. Statistical Analyses

The data was collected in an Excel spreadsheet, which was then imported into the Statistica 13 package in order to perform calculations. In this analysis, the data was compared between the cases of pregnancy-induced hypertension and the normotensive control group. Normality of data distribution in cases and the control group, was checked by the Shapiro-Wilk test. The Mann-Whitney U test was used for comparisons of continuous variables (distributions were not normally), and the Pearson chi-square test was used for categorical variables comparisons. *p*-value <0.05 was assumed to be significant.

Iron concentrations were compared between the case and control group using the Mann-Whitney U test; the values were presented as medians and 25%–75%, as means and SD (the distributions were not normally), *p*-value <0.05 was assumed to be significant. Only available measurements were taken into consideration.

We did individual matching. We chose the control normotensive group by matching cases of pregnancy-induced hypertension (in a 1: 3 ratio) in relation to the following criteria: Mother’s age, pre-pregnancy BMI and those who have never smoked. Due to the inability to select women at exactly the same age, we had to expand the selection by ± 2 years. The age in groups was similar, but not exactly the same (*p* = 0.907).

In order to assess the risk of pregnancy-induced hypertension, the uni- and multi-variate logistic regression was used and the whole cohort was divided into quartiles, based on the distribution of the iron concentrations (from 10–14 gestational week). The odds ratios of the disease for each quartile with regard to the highest reference quartiles (OR = 1.00 for Q4 quartile) and for the lowest Q1 quartile with regard to the higher quartiles were calculated. We calculated the odds ratios (OR and AOR) and 95% confidence intervals (CI). *p*-value was calculated using the Wald test, and value <0.05 was assumed to be significant. Graphs showing the risk profiles were presented.

The confounders were chosen from among the typical risk factors of the disease and/or microelement levels [9,11]. We identified matching variables (differing statistically insignificantly between groups). As confounders, we used the risk factors which differ statistically significantly between case and control groups: Pre-pregnancy BMI, gestational age during recruitment, gestational weight gain (GWG) for one week of pregnancy (calculated for whole gestation), family history of chronic hypertension, maternal education level <12 years.

Prognostic indicators of preeclampsia and gestational hypertension for iron concentrations were evaluated using ROC curves in the whole cohort (*N* = 484); best cut-off point, sensitivity, specificity and area under the curve (AUC) were determined. P-value was calculated using the Z test, and value <0.05 was assumed to be significant.

## 3. Results

The clinical characteristics of the cases of pregnancy-induced hypertension and normotensive controls are presented in Table 1. Between cases and their controls, differences were statistically insignificant in terms of: Mother’s age, primiparous, pack-years during recruitment and those who have never smoked, the number of assisted reproductive technology, the number of diabetes mellitus in the current pregnancy, multivitamins supplementation in II–III trimester. The average age at the conception in the group of cases was 35.1 years (range 19–45 years), and in the normotensive group was 35.1 years (range 22–45 years) (*p* = 0.907). The mean pre-pregnancy BMI was higher in the case group than in the normotensive group (*p* = 0.003). We found lower newborn birthweight (*p* = 0.0003) and lower gestational age et delivery (*p* = 0.011) in case group, compared to normotensive women.

The mean serum iron concentration was lower in the women subsequently developing pregnancy-induced hypertension compared to their matched normotensive women (*p* = 0.011). Available measurements only were taken into consideration (Table 1 and Table S1).

The socio-demographic characteristics in the normotensive controls and cases of pregnancy- induced hypertension are presented in Table 2. In the case group we found: More frequent occurrences of elementary and vocational education levels, and less often, declared a high financial status, compared to the matched normotensive control group.

The whole cohort was divided into quartiles based on the distribution of iron concentrations and the risk of pregnancy induced hypertension between quartiles was calculated (Table 3). In the lowest Q_1_ quartile (≤801.20 µg/L) the highest number of cases was found (45 cases among 120 women). The odds ratio (OR) of pregnancy induced hypertension was 2.19 (95% CI: 1.24–3.88, *p* = 0.007) for serum iron concentrations in the lowest Q_1_ quartile (≤801.20 µg/L) compared to the highest Q_4_ quartile (> 1211.75 µg/L) and the result was sustained after adjusted for all the accepted confounders (in multivariate logistic regression). Women in the higher Q_2_ quartile (801.20–982.33 µg/L) had a 17% lower risk, compared with those in the highest quartile.

**Figure 2:**
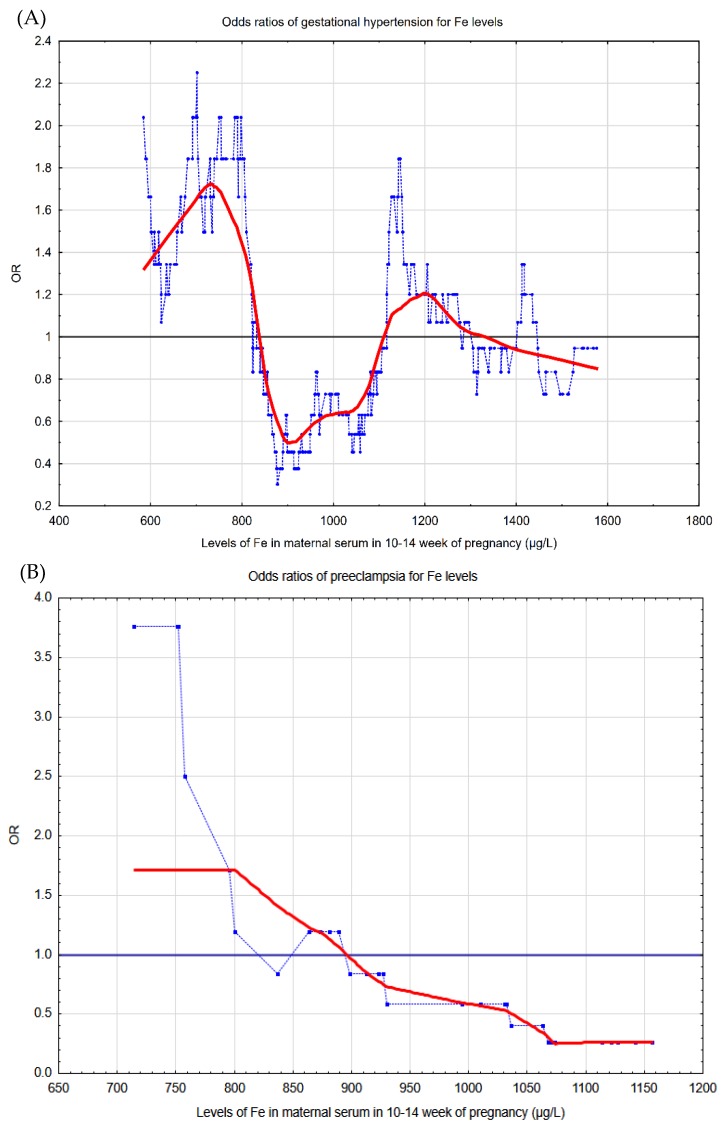
The graphical pictures of the risk profiles of (**A**) gestational hypertension and (**B**) preeclampsia for iron concentrations in 10–14 week of pregnancy. The graphs illustrate the changes in the odds ratios (OR), calculated on a sliding window with respect to the changes in the iron concentrations in serum in 10–14 pregnancy week: (**A**) the studied group covered 105 cases and 317 controls, and the window width adopted was 50 observations; (**B**) the studied group covered 15 cases and 45 controls, and the window width adopted was 30 observations. The (light blue) points correspond to the odds ratios of diseases in a window containing a fixed number of neighboring cases, whose center is for an iron concentration value. The (red) curve represents the risk profile smoothed with the Lowess method. The horizontal (black) line marks is the reference line for OR = 1.0; the points above this line indicate an increased risk, and the points below this line correspond to a reduction in risk.

Graphical pictures of the risk profiles of two forms of pregnancy-induced hypertension for serum iron concentrations (in 10–14 gestational week) can be seen in Figure 2. The pictures illustrate that the lowest iron concentrations were associated with the highest odds ratios of disease (OR > 1.00).

Prediction indicators of pregnancy-induced hypertension for serum iron concentrations (in 10–14 gestational week) are presented in Table 4. The ROC curve was evaluated for the whole cohort (*N* = 484). In predicting pregnancy-induced hypertension we obtained area under curve AUC = 0.578 (*p* = 0.013).

## 4. Discussion

We rated the relationship between serum iron concentrations in early healthy pregnancy, and the risk of pregnancy-induced hypertension. We found that the mean iron concentrations in 10–14 gestational weeks was lower in the case group compared to controls (*p* = 0.011). We found that women in the lowest quartile of iron (≤801.20 µg/L) had a 2.19-fold increase in pregnancy-induced hypertension risk compared with women in the highest quartile (>1211.75 µg/L). This result was sustained after adjusted for all the accepted confounders. Women in the higher Q_2_ quartile (801.20–982.33 µg/L) had a 17% lower risk, compared with those in the highest quartile.

The strength of our study was its prospective model in which one can assess whether iron can be a risk marker for pregnancy-induced hypertension; we studied the concentrations of iron in early healthy pregnancy. An advantage was the large number of cases of pregnancy-induced hypertension obtained in a single-center study. Case and control groups were well matched; the differences were statistically insignificant in terms of several risk factors of the disease. As confounders, we used the risk factors, which differ in statistically significance between the groups, but an impact of different confounders is possible. The groups were also compared in terms of socioeconomic characteristics and use of folic acid in trimester I, as well as multivitamins in trimester II-III. We used a new definition of pregnancy-induced hypertension, in which proteinuria is not a mandatory criterion of preeclampsia (yet it occurred in all the cases). Graphical pictures of the risk profiles of two forms of the disease (gestational hypertension and preeclampsia) were also a strength of the study.

We are aware of the limitations. We did not assess the concentrations of iron status markers (e.g., ferritin), but we believe that associations between iron and their markers are even more complex during pregnancy than in the general population. The participants declared fasting before blood samples were taken, but due to impaired peristalsis in pregnant women, this information may be of limited value. The participants of the study reported some data themselves (pre-pregnancy weight, smoking, alcohol), but the most important data on the current pregnancy came from the medical records, and all the information was verified during the observation.

In the literature, Basu et al. [18], in their prospective cohort study of 151 women with pre-pregnancy diabetes mellitus in single pregnancy, found insignificantly higher iron concentrations in the plasma in 12.2 +/–1.9 gestational week in 23 women who subsequently developed preeclampsia, compared to 24 women who remained normotensive. In the small pilot prospective study of 57 women, Tande et al. [19] found significantly lower serum iron concentrations in first trimester in the women who subsequently developed gestational hypertension, compared to normotensive women.

Retrospective studies are more numerous [16,17,23,24,25,26]. Fenzl et al. [25] showed significantly higher serum iron concentrations in 30 women with preeclampsia, compared to 37 healthy pregnant women. The same profile of results was found by Das et al. [26] in their study of 54 primiparas with pregnancy-induced hypertension, compared to normotensive primiparas. In contrast, Sarwar et al. [17] found significantly lower serum iron concentrations in 50 women with preeclampsia, compared to 58 women of the control group. The meta-analysis of 10 studies conducted by Liu et al. [16] provided significant evidence for higher serum/plasma iron concentrations in women with preeclampsia compared to healthy women in both the Asian and European population.

The discrepancies between various studies may be due to different research designs (retrospective or prospective studies) and differences in clinical methodology (population risk, size of groups, degree of matching and correction), as well as the biochemical methodology (determination of serum or plasma, various laboratory methods). In our prospective study, we evaluated iron concentrations in healthy women in early single pregnancy, and serum was the biological material. We stored serum samples at −80 °C, and to measure concentrations we used the method of mass spectrometry with inductively coupled plasma, which is considered a precise method. In our study, chronic diseases (except overweight and obesity) were excluded a-priori in the criteria of recruitment. In our study, the majority of cases developed gestational hypertension. We have well matched the groups to several recognized risk factors of the disease and iron status. Our study covered one geographical region of the country, which additionally matched the groups with respect to diet composition in the region and the same level of prenatal care.

We measured iron concentrations in the serum. However, in the assessment of the status of iron, different markers are used; e.g., serum ferritin and soluble transferrin receptor. In pregnancy, and especially in severe inflammations, the interpretation of iron concentration results may be difficult [1,27]. Ferritin is considered an indicator of iron storage concentrations, but at the same time is an acute phase protein and its level increases in inflammation [28,29]. Correlations of iron concentrations with hepcidin levels have also been studied in recent years [1,30].

In our study, in the case group we found a statistically significantly lower mean gestational age at recruitment and higher mean pre-pregnancy BMI. Other studies show that iron concentrations in pregnancy become lower in second trimester compared to first trimester [31]. In our study, we measured iron concentration in 10–14 gestational week, and the difference between case and control groups (11.55 vs. 12.25 weeks) was small (Table 1). In the literature, obesity increases the risk of pregnancy-induced hypertension [9,32]. Obesity and smoking reduce iron concentrations [20,21].

In our study, we compared several factors affecting iron levels and the development of this disease. (e.g., multivitamins supplementation in trimester II–III and socioeconomic factors). Some of the causes of iron deficiency mentioned include, the deficiency of folic acid, vitamin B12, and Vitamin A [27,33,34]. Research has shown that deficiencies of iron and vitamins (E, C, D, A, B, and folic acid) can induce inflammations in the placenta [35,36]. The involvement of iron, selenium, copper and zinc in the oxidative balance suggests the possibility of their association with the development of pregnancy-induced hypertension [18,37]. Iron deficiency in the mother causes an increase in the concentration of copper in the liver, but the mechanisms of their mutual interactions are complex [38,39]. Zinc ions get bound to metallothionines (MT) proteins that are involved in the transport of metals, among others iron [40].

The mechanisms, connecting the lowest serum iron level in 10-14 pregnancy week with the higher risk of pregnancy-induced hypertension, are complicated and can be connected with trophoblast development in this period. Pathomechanisms, leading to the development of preeclampsia/pregnancy-induced hypertension, are related to the complex molecular processes of placenta formation, in which numerous compounds are involved, including adhesion molecules, growth factors, immune cells, and transcription factors that perform key functions in protecting cells from the harmful effects of oxidative stress, enzymes, genes, apoptosis, and others [9,10,11,14,32]. It has been shown that, under the right conditions, low oxygen tension in the placenta, in 8–10 week of pregnancy, is accompanied by a low level of reactive forms of oxygen and nitrogen. Next, with the normal progression of trophoblast invasion, the blood flow in the placenta increases (in the 11–12 week of pregnancy, the oxygen tension was around 50 mmHg) and reactive forms of oxygen increases. However, the environment in the uterus is equipped with enzymes with antioxidant activity that protect cells from oxidative damage [36]. According to the main theory, abnormal invasion of trophoblasts in the walls of the spiral arteries (between 6–18 week of pregnancy) results in their insufficient re-modelling. This results in utero-placental high-resistance circulation, hypoxia and intensification of the production of reactive oxygen species, affecting apoptosis and the immune system, and intensification of the inflammatory response. The cascade of processes in placental and maternal circulation system is activated that leads to the damage of the vascular endothelium. The disturbance in endothelial homeostasis results, among others, in a vasoconstrictive and in an increase in blood pressure [9,10,11,14,15].

Iron plays a significant role inter alia in: Oxygen transport, in the production of ATP, in the synthesis of DNA, in preserving the function of mitochondria and in protecting cell structures against oxidative damage, in the activity of numerous enzymes, as well as in cell growth and proliferation [41,42]. Iron plays a catalyzing role in the production of reactive oxygen species in the Fenton and Haber-Weiss reactions [15]. The role of iron in the development of the placenta is not fully understood, but the discovery of the hypoxia-inducible factor (HIF) and its regulatory mechanisms also drew attention to the importance of iron. It has been shown that under oxygen deficient conditions or after using iron chelating agents (as an experimental model of hypoxia), hydroxylases cease to modify HIF-1a and increase HIF concentration, which in effect activates growth factors inhibiting the differentiation of trophoblast to the invasive phenotype [15,42]. Kadyrov et al. [43] showed that anemia was associated with increased apoptosis in the trophoblast.

Our main results give rise to the question of whether, iron should be compensated for in women in early pregnancy. There is considerable controversy in the assessment of iron supplementation in pregnant women. The meta-analysis conducted by Cantor et al. showed that there is no clear and consistent evidence for the beneficial effects of iron supplementation on the health of the mothers and the newborns [44]. It is known that the need for iron increases in pregnancy; anemia (resulting from iron deficiency) is a common problem in pregnant women [27]. It was found that iron deficiency correlates with some complications of pregnancy, e. g. with premature labor and low birthweight [8]. Studies have also shown that hemoglobin levels below 9.5–10.5 g/dL and levels above 13.0–13.5 g/dL are associated with a dramatic increase in adverse maternal and fetal results [27]. It was found that a high level of hemoglobin in women supplemented with iron was associated with an increase in the frequency of adverse effects, including preeclampsia [8,27]. In the study by Jirakittidul et al. [45] iron supplementation before 16 pregnancy week was associated with a significant increase in the risk of hypertension de novo after the 20th week of pregnancy. Ziaei et al. [46] found that the administration of iron at the beginning of the second trimester of pregnancy in women with a hemoglobin concentration of >13.2 g/dL increased the risk of pregnancy-induced hypertension. The results suggest that iron supplementation should be used in women with iron deficiency only.

## 5. Conclusions

In this prospective study, the lowest serum iron concentrations in 10–14 gestational week (≤801.20 µg/L), compared to iron in the highest quartile (>1211.75 µg/L) were associated with significantly two-fold higher risk of pregnancy-induced hypertension. This result was sustained after being adjusted for all the accepted confounders.

The iron concentrations in Q_2_ quartile (801.20–982.33 µg/L) were associated with the lowest number of cases of pregnancy-induced hypertension. Women in the Q_2_ quartile had a 17% lower risk, compared with those in the highest quartile. However, only well-designed randomized trials can answer the question of whether achieving optimal concentrations of iron can affect the risk of disease, and what is the optimal concentration for specific gestational age.

In our opinion, the measurement of serum iron concentration in early pregnancy can be included in diagnostics for identifying women at risk of pregnancy-induced hypertension.

In our opinion, serum iron concentration can be a risk marker of pregnancy induced hypertension, however the future research is needed.

## Figures and Tables

**Table 1:** The clinical characteristics of the normotensive controls and cases of pregnancy-induced hypertension.

Characteristics	Controls (*n* = 363) *	Cases (*n* = 121) *	*p* **
	N (%) or Mean (SD); Median	N (%) or Mean (SD); Median	
**Basic:**			
Maternal age (years)	35.05 (3.99); 36.00	35.05 (4.21); 36.00	0.907
Maternal age (range)	(22–45)	(19–45)	
Pre-pregnancy BMI (kg/m^2^)	25.03 (4.40); 24.38	26.76 (5.37); 26.47	0.003
Pre-pregnancy BMI (range)	(16.52–39.41)	(18.17–42.91)	
Pre-pregnancy BMI ≥30 kg/m^2^	48 (13.22%)	34 (28.10%)	0.0002
Gestational age at recruitment (weeks)	12.25 (0.80); 12.00	11.55 (0.82); 11.00	1.97 × 10^−16^
Gestational age at recruitment (range)	(10–14)	(10–14)	
Primiparous	141 (38.84%)	56 (46.28%)	0.149
Prior PE ***	2 (0.55%)	13 (10.74%)	<0.001
Pre-term delivery in history	19 (5.23%)	7 (5.79%)	0.816
ART	18 (4.96%)	11 (9.09%)	0.097
Women who had never smoked	302 (83.20%)	92 (76.03%)	0.080
Pack-years during recruitment	19.25 (32.51); 6.63	21.21 (32.26); 8.75	0.748
**Vitamin supplementation**			
Folic acid supplementation in I trimester	126 (34.71%)	22 (18.18%)	0.0006
Multivitamin supplementation in II-III trimester	184 (50.69%)	50 (41.32%)	0.074
Microelement concentrations (µg/L) ****			
Iron	1043.85 (338.77); 995.66	948.86 (333.73); 908.27	0.011
Selenium	62.89 (8.70); 62.02	57.51 (6.54); 57.40	<0.00001
Copper	1767.53 (338.67); 1746.67	1698.33 (298.37); 1671.44	0.059
Zinc	628.03 (174.57); 607.49	610.19 (87.58); 607.66	0.690
**Outcomes**			
PE	-	15	-
GDM	73 (20.11%)	23 (19.01%)	0.792
Gestational age at delivery (weeks)	38.71 (1.77); 39.0	37.99 (2.62); 39.0	0.011
Delivery <37 week	23 (6.34%)	16 (13.22%)	0.016
Newborn birthweight (g)	3385.28 (546.81); 3400.00	3113.06 (785.36); 3150.00	0.0003
Birthweight <10 centiles	21 (5.79%)	22 (18.18%)	0.00003

* Normotensive controls and cases of pregnancy-induced hypertension (PIH); ** The Mann-Whitney U test was used for comparisons of continuous variables, *p*-value <0.05 was assumed to be significant (medians were compared); ** The Pearson chi-square test was used for categorical variables comparisons (*p*-value <0.05 was assumed to be significant); *** PE: preeclampsia; ART: assisted reproductive technology; GDM Gestational Diabetes Mellitus; **** in serum from 10–14 gestational week.

**Table 2:** The socio-demographic characteristics in the normotensive controls and cases of pregnancy-induced hypertension.

Socio-Demographic Characteristics	Controls (*n* = 363) *	Cases (*n* = 121) *	*p* **
	*n* (%)	*n* (%)	
**Education Levels (Available data, *n*)**	305	105	0.042
Higher	201 (65.90%)	57 (54.29%)	
Secondary	76 (24.92%)	28 (26.67%)	
Vocational	26 (8.53%)	17 (16.19%)	
Elementary	2 (0.66%)	3 (2.86%)	
Financial status (available data, *n*) ***	141	63	0.002
(lower levels)	14 (9.93%)	5 (7.94%)	
(medium level)	32 (22.70%)	26 (41.27%)	
(higher levels)	95 (67.38%)	32 (50.79%)	
**Place of residence (available data, *n*)**	362	120	0.585
Country	110 (30.39%)	30 (25.00%)	
Town <50,000 of residence	104 (28.73%)	35 (29.17%)	
Big city >50,000 of residence	148 (40.88%)	56 (46.67%)	

* Normotensive controls and cases of pregnancy-induced hypertension (PIH); ** The Pearson chi-square test was used for variables comparisons (*p*-value <0.05 was assumed to be significant); *** in the Lickert’s Scale.

**Table 3:** The odds ratios of pregnancy-induced hypertension for serum Cu levels, in the ideal subgroup.

Quartile	Iron (µg/L)!	Risk of Pregnancy-Induced Hypertension							
		Cases	Controls	OR *	(CI 95%)	*p* ***	AOR **	(CI 95%)	*p* ***
Q_1_	217.55–801.20	45	75	2.19	(1.24–3.88)	0.007	1.98	(1.01–3.90)	0.048
Q_2_	801.20–982.33	22	99	0.83	(0.65–2.32)	0.519	0.92	(0.53–2.23)	0.816
Q_3_	982.33–1211.75	27	93	1.06	(0.58–1.95)	0.850	1.43	(0.72–2.85)	0.310
Q_4_	1211.75–2806.24	26	95	1			1		

! Iron concentrations were measured in serum from 10–14 gestational week and border values are included in the lower quartile; Cases of pregnancy induced hypertension and Controls: normotensive women; * OR: crude odds ratio calculated in univariate logistic regression; AOR **: adjusted odds ratio calculated in multivariate logistic regression, after adjusting for the pre-pregnancy BMI, gestational age at recruitment, gestational weight gain for week of whole gestation, family history of chronic hypertension, maternal education <12 years; CI: confidence intervals; *** *p* < 0.05 was assumed to be significant

**Table 4:** Prediction indicators of pregnancy induced hypertension for iron concentrations, calculated using and ROC curve.

Disease	Prediction Indicators of Disease for Iron Concentrations *				
	Cut-Off Point (µg/L)	AUC	*p* **	Sensitivity	Specificity
Pregnancy induced hypertension	843.21	0.578	0.013	45.8%	74.0%

* Iron concentrations were measured in serum from 10–14 gestational week (µg/L); ** *p*-value obtained using the Z test; *p* < 0.05 was assumed to be significant.

## Supplementary Materials

The following are available online at https://www.mdpi.com/2072-6643/11/5/1086/s1, Table S1: Complete characteristics of serum iron (Fe) levels in groups.
