# Supplementary material for: Can Serum Iron Concentrations in Early Healthy Pregnancy Be Risk Marker of Pregnancy-Induced Hypertension?
Source: Nutrients. 2019 May 16;11(5):1086. doi: 10.3390/nu11051086 (PMC6566422; doi:10.3390/nu11051086)
Supplement: Supplementary file 1 [file nutrients-11-01086-s001.zip › nutrients-485978-SI.pdf]

Table S1. Complete characteristics of serum iron (Fe) levels in groups

Groups	n	Serum Fe levels *							
		**Mean**	**SD**	**Min.**	**Max.**	**Q25**	**Median**	**Q75**	**p*** **
**Gestational hypertension (GH)**									
Controls **	317	1044.08	340.74	314.76	2806.24	838.81	994.68	1237.01	
Cases of GH	105	972.28	336.03	217.55	1884.86	739.10	930.79	1181.96	0.096
**Preeclampsia (PE)**									
Controls **	45	1042.21	328.31	413.94	1858.50	863.86	1031.03	1211.75	
Cases of PE	15	784.90	273.83	483.76	1405.18	578.49	676.78	1067.56	0.008

* Iron (Fe) levels were measured in serum from 10-14 gestational week (μg/L); ** Normotensive controls; *** p- value obtained using the Mann-Whitney U test; $p < 0.05$ was assumed to be significant
